# Multi-Omics Data Fusion via a Joint Kernel Learning Model for Cancer Subtype Discovery and Essential Gene Identification

**DOI:** 10.3389/fgene.2021.647141

**Published:** 2021-03-04

**Authors:** Jie Feng, Limin Jiang, Shuhao Li, Jijun Tang, Lan Wen

**Affiliations:** ^1^School of Computer Science and Technology, College of Intelligence and Computing, Tianjin University, Tianjin, China; ^2^School of Computational Science and Engineering, University of South Carolina, Columbia, SC, United States; ^3^Key Laboratory of Systems Bioengineering (Ministry of Education), Tianjin University, Tianjin, China; ^4^Changsha Municipal Center of Disease Control, Changsha, China

**Keywords:** cancer subtype, kernel PCA, spectral clustering, survival analysis, GSEA

## Abstract

The multiple sources of cancer determine its multiple causes, and the same cancer can be composed of many different subtypes. Identification of cancer subtypes is a key part of personalized cancer treatment and provides an important reference for clinical diagnosis and treatment. Some studies have shown that there are significant differences in the genetic and epigenetic profiles among different cancer subtypes during carcinogenesis and development. In this study, we first collect seven cancer datasets from the Broad Institute GDAC Firehose, including gene expression profile, isoform expression profile, DNA methylation expression data, and survival information correspondingly. Furthermore, we employ kernel principal component analysis (PCA) to extract features for each expression profile, convert them into three similarity kernel matrices by Gaussian kernel function, and then fuse these matrices as a global kernel matrix. Finally, we apply it to spectral clustering algorithm to get the clustering results of different cancer subtypes. In the experimental results, besides using the *P*-value from the Cox regression model and survival analysis as the primary evaluation measures, we also introduce statistical indicators such as Rand index (RI) and adjusted RI (ARI) to verify the performance of clustering. Then combining with gene expression profile, we obtain the differential expression of genes among different subtypes by gene set enrichment analysis. For lung cancer, GMPS, EPHA10, C10orf54, and MAGEA6 are highly expressed in different subtypes; for liver cancer, CMYA5, DEPDC6, FAU, VPS24, RCBTB2, LOC100133469, and SLC35B4 are significantly expressed in different subtypes.

## Introduction

Cancer is the important leading cause of death in the world and is responsible for an estimated 9.6 million deaths in 2018. Unlike most other diseases, cancer is not a sort of single disease but is a group of diseases involving abnormal cell growth with the potential to invade or spread to other parts of the body. In the same type of cancer, patients usually have the same or similar external appearances, but in most cases, universal drugs and universal treatment methods do not produce good prognosis in all cases. The multiple sources of cancer determine its multiple causes, and the same cancer can be composed of many different subtypes. The discovery and identification of cancer subtypes are a key part of personalized cancer treatment and provide an important reference for clinical diagnosis and treatment (de Kruijf et al., 2013).

The Cancer Genome Atlas (TCGA) is the largest open cancer genome database to date initiated by the US government, which aims to catalog and discover major cancer-causing genome alterations in large cohorts of over 30 human tumors through large-scale genome sequencing and integrated multidimensional analyses. It covers a variety of omics expression data including genomics, transcriptomics, copy number variation, DNA methylation, proteomics, and clinical information of follow-up cases (Tomczak et al., 2015; Jiang et al., 2019a), which provide great support for the detection of cancer subtypes by computational methods.

Recently, many methods for cancer subtypes recognition and marker extraction have been proposed (Yeoh et al., 2002; Lapointe et al., 2004; Figueroa et al., 2010; Yang et al., 2017a; Pan et al., 2019). Some models are based on single expression data, including gene expression (Yeoh et al., 2002; Lapointe et al., 2004), microRNA (miRNA) expression (Yang et al., 2017a,b; Liu and Yang, 2018), copy number variation (Pan et al., 2019), and DNA methylation (Figueroa et al., 2010). Lapointe et al. (2004) identified three subclasses of prostate tumors based on distinct patterns of gene expression. Yang et al. (2017a) clustered miRNAs based on Fisher linear discriminant analysis (FDA), using representative cluster member combinations as potential biomarkers. Pan et al. (2019) used copy number variation, a biomarker more likely to be used for cancer diagnosis than mRNA biomarkers, to further reveal differences between various breast cancer subtypes. Figueroa et al. (2010) examined the methylation profiles of 344 patients with acute myeloid leukemia (AML). Clustering of these patients by methylation data segregated patients into 16 groups. Five of these groups defined new AML subtypes. Also, there are methods to analyze and predict cancer subtypes by considering multiple expression data (Shen et al., 2009; Wang et al., 2014; Ge et al., 2016; Jiang et al., 2019b). The iCluster is a latent variable model-based clustering algorithm proposed by Shen et al. (2009). It uses multiple sources of data for integrated analysis to identify tumor subtypes. Similarity network fusion (SNF) is a network fusion method integrating multicomponent data, which was proposed by Wang et al. (2014). SNF builds a similar network of sample pairs on different histological data (gene, methylation, and miRNA) and then integrates the network to predict cancer subtypes.

Furthermore, due to the high dimensionality of research data, we need to find effective and suitable dimensionality reduction methods. Some methods, such as principal component analysis (PCA) and non-negative matrix factorization (NMF), have been used to combine clustering algorithms (Alter et al., 2000; Holter et al., 2000; Brunet et al., 2004). However, for the high-dimensional and non-linear gene data, the performance is not always good. In order to better handle these, we consider a non-linear version of PCA, kernel PCA (Schölkopf et al., 1998), which introduces a non-linear mapping function that can map data in the raw space to high-dimensional space. It can make the distribution of all mapped data linearized and simplified in high-dimensional space, and then PCA can be used to construct features.

In this study, inspired by SNF, we combine gene expression profile with isoform expression profile and DNA methylation expression data. We propose a novel method: first, take kernel PCA to extract features for each profile, then convert them into three similarity kernel matrices, and fuse them into one. Finally, we apply it to spectral clustering algorithm to get the clustering results of different cancer subtypes.

## Materials and Methods

**FIGURE 1:**
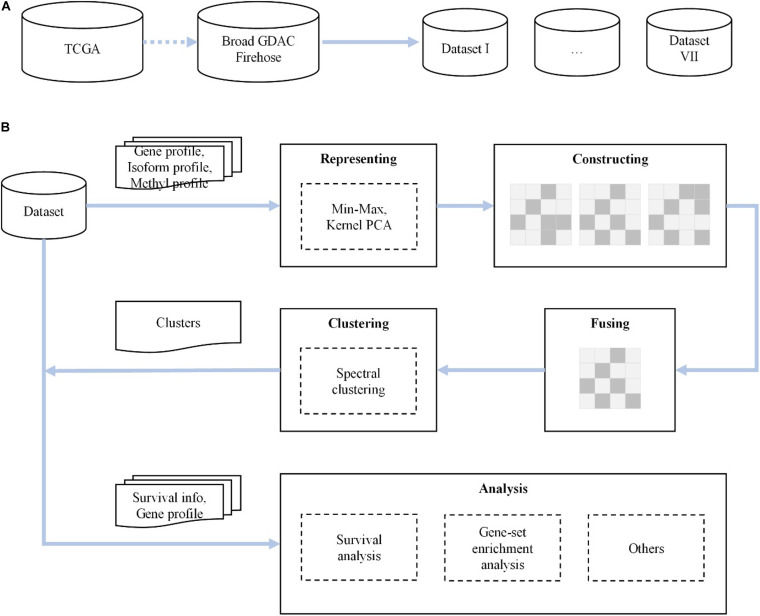
The flowchart of our novel method. **(A)** We first collect and check seven cancer datasets from The Cancer Genome Atlas (TCGA) dataset of the Broad Institute GDAC Firehose. **(B)** For each dataset, after representing, constructing, fusing, and clustering for gene, isoform, and DNA methylation data, we carry out survival analysis, gene set enrichment analysis, and others.

We propose a novel method for analyzing various cancer subtypes. First, we rescale the raw expression data by min–max normalization and reduce the dimensionality of data via kernel PCA, with a minimal loss of information. Then, in each cancer dataset, based on the expression of gene profile, isoform profile, and methylation data, we construct three similarity kernel matrices through the Gaussian kernel function and fuse them into a global similarity expression matrix. Finally, the integrated similarity kernel matrix is fed to spectral clustering, and the predictive clusters are identified. The flowchart of our proposed method is shown in Figure 1.

### Data Sources

In our study, all research data are collected from the Broad Institute GDAC Firehose^1^ (Center BITGDA, 2016). Firehose is an analytical infrastructure created at the Broad Institute based on the data of TCGA project (Tomczak et al., 2015), which provides genome-scale transcriptome data for various cancers and different levels of processed data for cancer analysis. Firehose gives a corresponding visual web platform, Firebrowse^2^, which can easily access TCGA open access layer data. This greatly lowers the threshold for experimenters to operate the TCGA database and also makes the data for analyzing as consistent as possible. Here, we extract seven common cancer datasets: BLCA, BRCA, COAD, KIDNEY (KICH&KIRC&KIRP), LIHC, LUNG (LUAD&LUSC), and STAD. For each cancer dataset, it consists of gene expression information (gdac_rnaseqv2_genes_RSEM_normalized_Level_3, 2016-02- 18), isoform expression information (gdac_rnaseqv2_isoforms_ RSEM_normalized_Level_3, 2016-02-18), DNA methyla-tion expression information (gdac_Methylation_Preprocess_ mean_Level_3, 2016-02-18), and corresponding clinical information (gdac_Clinical_Pick_Tier1_Level_4, 2016-02-18). The clinical data are used in subsequent survival analyses, while the other three expression profiles are used to construct a suitable global similarity kernel matrix. We check redundant cases (reserve cases with number 01–09) in the four profiles at each cancer datasets and extract all valid cases that contain the above expression information. And then, we obtain the experimental input data for cluster analysis. A summary of our datasets is shown in Table 1.

**TABLE 1:** Description of seven datasets.

Datasets	No. samples	Gene	Isoform	Methylation
BRCA	780	20,531	73,599	20,106
COAD	275	20,531	73,599	20,116
KIDNEY	658	20,531	73,599	20,119
LUNG	824	20,531	73,599	20,116
STAD	372	20,531	73,599	20,101
BLCA	408	20,531	73,599	20,109
LIHC	371	20,531	73,599	20,105

### Data Representation

#### Min–Max Normalization

Since we collect high-quality, standardized datasets directly from Firehose, the size of the data values can intuitively reflect the expression abundance of gene, isoform, or methylation. However, to eliminate the influence of digital distribution in three expression profiles and make them fused reasonable, we use min–max normalization to rescale the values. The general formula for a min–max of [0, 1] is defined as Eq. 1:

(1) x=′x-min(x)max(x)-min(x)

where *x* is an original expression value and *x*′ is the rescaled value.

#### Kernel Principal Component Analysis

The kernel PCA is a method for performing a non-linear form of PCA proposed in Schölkopf et al. (1998). Through using kernel PCA, the dimensionality of complex, non-linear features can be reduced effectively. Kernel PCA transforms the raw linear input space *R* into a high-dimensional feature space *F* by using some non-linear mapping, like a dot product matrix defined as Eq. 2:

(2) $$K\left(x_{i},x_{j}\right)=\left\langle \Phi \left(x_{i}\right),\Phi \left(x_{j}\right)\right\rangle$$

and calculates the principal components in *F*. Then compute projections onto the eigenvectors obtained by diagonalizing *K* to extract the principal components corresponding to the *k* of *K* (Schölkopf et al., 1998; Devi et al., 2014). In this study, we use the kernel PCA method and take polynomial kernel defined as Eq. 3:

(3) $$K_{poly}\left(x_{i},x_{j}\right)={\left(x_{i}^{T}x_{j}+1\right)}^{3}$$

as the non-linear mapping. We adopt the default parameter, *xi* and *xj* are the expression vector of *i-*th case and *j*-th, and all the non-zero components are preserved. After performing the above rescale and reduction on the gene, isoform, and methylation expression profiles in the dataset, we gain the necessary input to construct a similarity kernel matrix.

### Similarity Kernel Matrix

#### Kernel Construction

The kernel methods map data points into possibly high-dimensional feature space, where the distribution of all mapped data is linearized and simplified (Vert et al., 2004; Mei and Fei, 2010). Assume mapping function Φ(*x*), the computation of the inner product ⟨Φ(*x*_*i*_), Φ(*x*_*j*_)⟩ in the high-dimensional feature space *F* can be implemented in the original space *R* using kernel trick, *K*(*x*_*i*_, *x*_*j*_) = ⟨Φ(*x*_*i*_), Φ(*x*_*j*_)⟩, such that no explicit mapping function or even explicit feature representation is required. The size of the matrix used to represent the profile of *N* cancer cases is always *N* by *N.* This allows us to comprehensively consider the expression of three profiles for one specific cancer, which perform a more accurate cluster analysis (Vert et al., 2004). Here, we use the Gaussian kernel and the adjusted parameter γ, as Eq. 4:

(4) $$K_{gaussian}\left(x_{i},x_{j}\right)=exp\left(-\gamma {||x_{i}-x_{j}||}^{2}\right)$$

#### Kernel Fusion

Data fusion from multidimensional expression profiles has been shown to produce better results than considering a single expression information. Jiang et al. (2019b) and Li et al. (2020) have researched multi-omics data fusion and achieved good results. We fuse three different expression profiles (gene, isoform, and methylation) to construct a global similarity kernel matrix for each cancer. Therefore, we integrate three Gaussian kernel matrices. In our example, we adopt an average fusion strategy as Eq. 5:

(5) $$K_{fuse}=\frac{1}{3}\left(K_{gene},K_{isoform},K_{methyl}\right)$$

where *K*_*gene*_, *K*_*isoform*_, and *K*_*methyl*_ represent the similarity kernel matrix constructed by gene, isoform, and methylation expression profiles, respectively.

### Spectral Clustering

Spectral clustering is a clustering method based on graph theory algorithm; the basic idea is to use the similarity matrix of the samples to obtain the feature vector of the feature decomposition for cluster analysis (Von Luxburg, 2007). Because of its excellent algebraic graph foundation, it can get a global loose solution for complex cluster structure (Jia et al., 2014). We use it as the core algorithm for cluster analysis. The process of spectral clustering algorithm is taken as follows. First, based on *K*_*fuse*_, calculate the Laplacian matrix *L.* Then, construct the normalized Laplacian matrix *D*^−1/2^*LD*^−1/2^. *D* is a diagonal matrix whose diagonal element is the sum of the row elements of *K*_*fuse*_. And compute the eigen vectors *y* corresponding to the eigen values of *D*^−1/2^*LD*^−1/2^. The matrices composed of corresponding eigen vectors *y* are standardized on a row basis to form the *N*_*case*_ × *N*_*feature*_ feature matrix *Y*. Finally, each row in *Y* is taken as a sample, which is clustered by discrete method to obtain cluster partition *C*(*C*_1_, *C*_2_, …, *C*_*j*_). Each partition will represent a cancer subtype. The whole process of spectral clustering can be transformed into solving the optimization problem as Eq. 6:

(6) $$\begin{array}{c} mintr\left(Y^{T}D^{-\frac{1}{2}}LD^{-\frac{1}{2}}Y\right)\\ s.t.Y^{T}Y=I \end{array}$$

where *Y* is the eigen matrix for the eigen values of *D*^−1/2^*LD*^−1/2^, *D* is the degree matrix of *K*_*fuse*_, and *L* is the Laplacian matrix of *K*_*fuse*_.

## Results

In this section, we evaluate and compare the performance of our proposed method in multiple dimensions, using *P*-values and survival curves as the primary criteria and taking indexes such as RI and ARI into consideration. Finally, through gene set enrichment analysis (GSEA), some key genes supporting each subtype are obtained and displayed using heat maps and boxplots.

### Evaluation Novel Method

We use the *P*-value of Cox regression model to evaluate the performance of several key steps of the proposed method (Pölsterl et al., 2015, 2016a,b). It includes applying kernel PCA to reduce the original data dimension, using similarity kernel fusion strategy to obtain feature input, and employing spectral clustering as the core clustering method to obtain the final clustering result. We calculate the *P*-values for the clusters on the seven datasets. A lower *P*-value indicates a more significant result. Here, we use 0.05 as the threshold for evaluation.

#### Performance of Kernel Principal Component Analysis

The kernel PCA is a non-linear version of PCA widely used in linear dimensionality reduction methods. Using kernel PCA, the dimensionality of complex, non-linear features can be reduced effectively. For the features with tens of thousands of dimensions in the original data, it can be reduced to only a few hundred. We compare all datasets, with and without kernel PCA, and the resulting *P*-values are shown in Table 2. According to the data in Table 2 (the number of clusters in the table is the optimal results with the lowest *P*-values), kernel PCA greatly improves the performance of the method and makes the results more reliable and stable.

**TABLE 2:** Results with or without kernel principal component analysis (KPCA).

Datasets	*P*-value without KPCA	*P*-value with KPCA
BRCA(4)	0.137	9.71e–06
COAD(10)	0.465	2.39e–03
KIDNEY(9)	0.381	7.15e–04
LUNG(4)	0.386	9.06e–03
STAD(6)	0.977	2.35e–03
BLCA(9)	0.290	1.88e–05
LIHC(7)	0.038	1.23e–07

#### Performance of Fusion Strategy

Here, we compare the performance of using a single kernel directly with the use of a kernel fusion strategy on seven datasets, and the results are shown in Table 3. After using similarity kernel fusion, the *P*-values on seven datasets have been significantly improved. And for the dataset LIHC, although the performance on single kernel has already performed well, kernel fusion will further enhance the clustering results. We therefore conclude that the strategy for similarity kernel fusion is necessary.

**TABLE 3:** *P*-values of taking single kernel and fused kernel.

Datasets	Gene	Isoform	Methyl	Fusion
BRCA(4)	0.011	0.342	0.755	9.71e–06
COAD(10)	0.024	0.693	0.254	2.39e–03
KIDNEY(9)	0.057	0.475	0.116	7.15e–04
LUNG(4)	0.667	0.565	0.142	9.06e–03
STAD(6)	0.063	0.552	0.252	2.35e–03
BLCA(9)	0.090	0.116	0.192	1.88e–05
LIHC(7)	0.001	0.004	0.071	1.23e–07

#### Comparing With Other Methods

We compare the results with those of the method of Li et al. (2020). As shown in Table 4, we find that using kernel PCA for feature reduction, taking weighted fusion strategies instead of complex SKF, and finally generating clustering results from spectral clustering have comparable reliability and stability.

**TABLE 4:** Performance between Li’s and our method.

Datasets	Li’s method	Our method
BRCA(4)	1.12e–05	9.71e–06
COAD(10)	1.12e–07	2.39e–03
KIDNEY(9)	1.80e–02	7.15e–04
LUNG(4)	1.59e–06	9.06e–03
STAD(6)	2.00e–03	2.35e–03
BLCA(9)	–	1.88e–05
LIHC(7)	–	1.23e–07

### Clustering Analysis

The Rand index (RI) (Rand, 1971) is an indicator for evaluating clustering performance in statistics, by measuring the similarity between two data clusters. However, a problem with the index is that the expected value of the RI of two random partitions cannot take a constant value (Steinley, 2004). The adjusted RI proposed by Yeung and Ruzzo (2001), which is the corrected-for-chance version of the RI, can effectively avoid RI’s insufficient. We measure both the LUNG and KIDNEY datasets and compare the results obtained by Li et al. (2020). The results are shown in Table 5. From the indicators such as RI and ARI, our clustering is more stable, and the effect is much better than Li’s in KIDNEY and LUNG. Especially on LUNG, although we get a higher *P*-value, we have a 28% advantage on the ARI.

**TABLE 5:** Rand index (RI) and adjusted Rand index (ARI) on two datasets between Li’s and our method.

Datasets	RI of Li	Our RI	ARI of Li	Our ARI
KIDNEY(9)	0.59	0.66	0.07	0.21
LUNG(4)	0.5	0.64	0	0.28

### Survival Analysis

**FIGURE 2:**
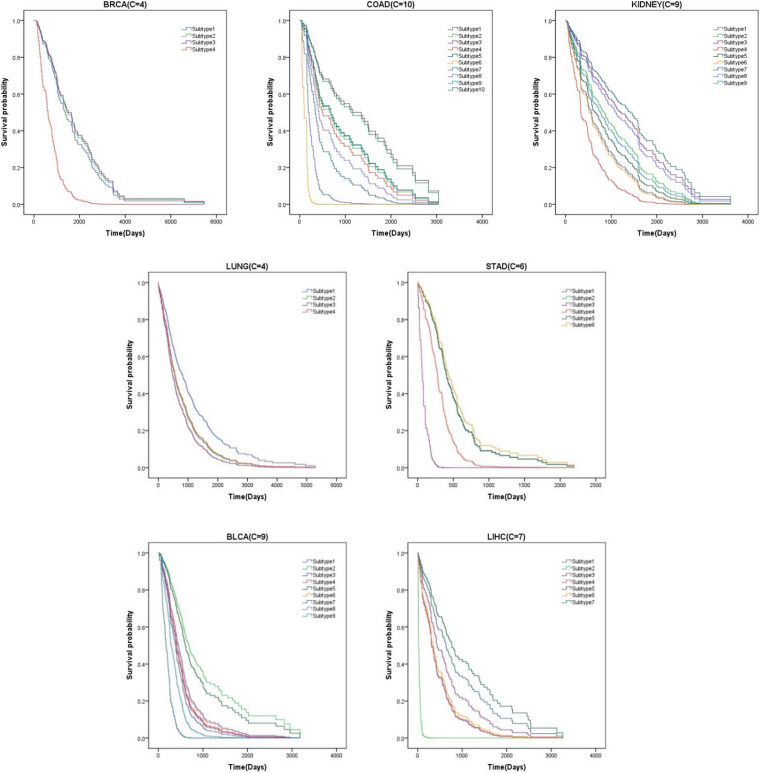
Survival curves of various subtypes for seven cancer datasets.

Survival analysis is a branch of statistics that analyzes the expected duration until an event occurs, such as the death of a cancer patient. We find that patients with subtype 2 of liver cancer (LIHC), subtype 6 of colon cancer (COAD), and subtype 3 of stomach cancer (STAD) have higher mortality. More attention should be paid to these patients. We also see that the average survival time of breast cancer patients (BRCA) and lung cancer patients (LUNG) is longer than that of others. It indicates that these cluster results can be used to guide clinical treatment. The survival curves of all datasets are shown in Figure 2.

### Gene Set Enrichment Analysis

**FIGURE 3:**
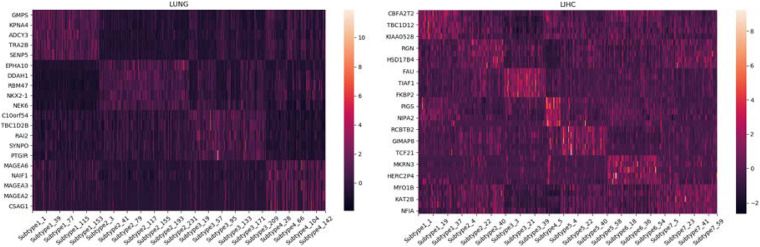
Differential expression of some essential genes in different subtypes on LUNG and LIHC.

GSEA (Mootha et al., 2003; Subramanian et al., 2005; Huang et al., 2020) is an analysis method for genome-wide expression profile chip data, which compares genes with a predefined set of genes. Synthesize the existing information base of gene location, nature, function, biological significance, etc., to build a molecular tag database (MSigDB), in which known genes are identified by chromosomal location, established gene set, and model sequence. Tumor-related gene set and GO gene set and other functional gene sets are grouped and classified. By analyzing the gene expression profile data, we can understand their expression status in a specific functional gene set and whether this expression status has some statistical significance. In this paper, we use Broad Institute’s offline analysis software GSEA_4.0.2, and C4 collection (cancer gene neighborhoods and cancer modules), provided by Broad Institute in the Molecular Signatures Database (MSigDB), which is a computational gene set defined by mining large collections of cancer-oriented microarray data. We analyze the LUNG and LIHC datasets, and we collect the expression data of genes with higher scores on different subtypes. The heat maps drawn are shown in Figure 3.

### Essential Gene Analysis

**FIGURE 4:**
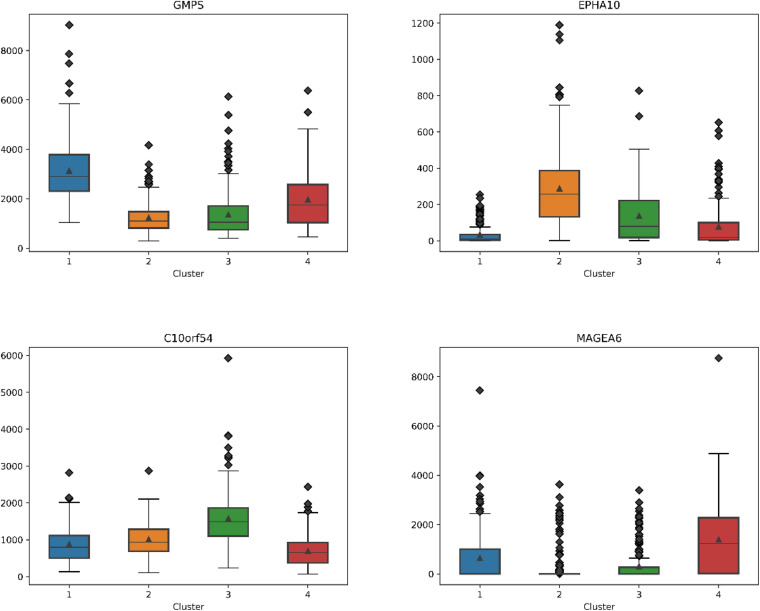
The boxplots for essential genes supporting each subtype on LUNG.

**FIGURE 5:**
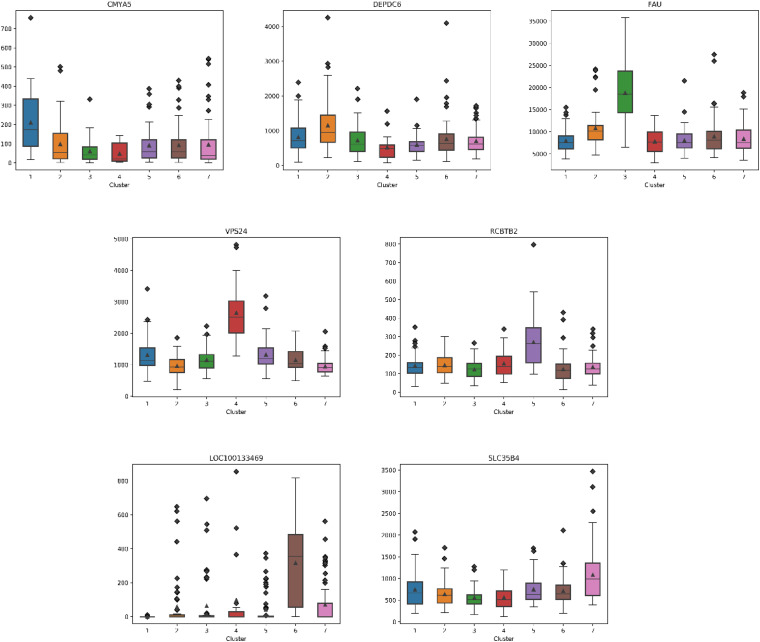
The boxplots for essential genes supporting each subtype on LIHC.

For each subtype on the datasets LUNG and LIHC, we select the essential gene that can highly distinguish the subtypes. According to the expression of each gene on its dataset, we obtain the box diagrams as shown in Figures 4, 5. We find that GMPS, EPHA10, C10orf54, and MAGEA6 are highly expressed in different subtypes on the dataset LUNG; and CMYA5, DEPDC6, FAU, VPS24, RCBTB2, LOC100133469, and SLC35B4 are significantly expressed in different subtypes on the dataset LIHC, respectively.

## Conclusion

In this paper, we propose a model for accurately predicting cancer subtypes. First, we collect seven cancer datasets from Firehose website, which contained three kinds of expression data (gene expression, isoform expression, and methylation expression). Then we construct three similar kernels for three kinds of expression data, respectively, and we fuse the three kernels into the global one. Finally, the cancer subtypes are discovered by spectral clustering. We take *P*-value as the overall evaluation criterion, combining with survival curve analysis and GSEA.

In the future, we will also try other machine learning methods or deep learning methods (Kong and Yu, 2018; Ding et al., 2019a,b; Shen et al., 2019; Gao et al., 2020; Lee et al., 2020; Wang et al., 2020), to deal with the problem of small samples and large features of cancer data and predict cancer subtypes more accurately.

## Data Availability Statement

The original contributions presented in the study are included in the article/Supplementary Material, further inquiries can be directed to the corresponding author/s.

## Author Contributions

JF and SL conceived and designed the experiments. JF and LJ performed the experiments and analyzed the data. LW and LJ wrote the manuscript. JT supervised the experiments and reviewed the manuscript. All authors contributed to the article and approved the submitted version.

## Conflict of Interest

The authors declare that the research was conducted in the absence of any commercial or financial relationships that could be construed as a potential conflict of interest.

## Footnotes

1 https://gdac.broadinstitute.org

2 http://firebrowse.org/

## References

[B1] Alter O. Brown P. O. Botstein D. (2000). Singular value decomposition for genome-wide expression data processing and modeling. *Proc. Natl. Acad. Sci.U.S.A.* 97 10101–10106. 10.1073/pnas.97.18.10101 10963673 PMC27718

[B2] Brunet J.-P. Tamayo P. Golub T. R. Mesirov J. P. (2004). Metagenes and molecular pattern discovery using matrix factorization. *Proc. Natl. Acad. Sci.U.S.A.* 101 4164–4169. 10.1073/pnas.0308531101 15016911 PMC384712

[B3] Center BITGDA (2016). *Analysis-Ready Standardized TCGA Data From Broad GDAC Firehose 2016_01_28 run: Dataset.* Cambridge, MA: Broad Institute of MIT and Harvard, 10.7908/C11G0KM9

[B4] de Kruijf E. M. Engels C. C. van de Water W. Bastiaannet E. Smit V. T. van de Velde C. J. (2013). Tumor immune subtypes distinguish tumor subclasses with clinical implications in breast cancer patients. *Breast Cancer Res. Treat.* 142 355–364. 10.1007/s10549-013-2752-2 24197659

[B5] Devi H. S. Thounaojam D. M. Laishram R. (2014). An approach to illumination and expression invariant multiple classifier face recognition. *Int. J. Comput. Appl.* 975:8887. 10.5120/15959-5335 7911558

[B6] Ding Y. Tang J. Guo F. (2019a). Identification of drug-side effect association via multiple information integration with centered kernel alignment. *Neurocomputing* 325 211–224. 10.1016/j.neucom.2018.10.028

[B7] Ding Y. Tang J. Guo F. (2019b). Identification of drug-target interactions via fuzzy bipartite local model. *Neural Comput. Appl.* 32 10303–10319. 10.1007/s00521-019-04569-z

[B8] Figueroa M. E. Lugthart S. Li Y. Erpelinck-Verschueren C. Deng X. Christos P. J. (2010). DNA methylation signatures identify biologically distinct subtypes in acute myeloid leukemia. *Cancer cell* 17 13–27. 10.1016/j.ccr.2009.11.020 20060365 PMC3008568

[B9] Gao J. Lyu T. Xiong F. Wang J. Ke W. Li Z. (2020). “MGNN: a multimodal graph neural network for predicting the survival of cancer patients,” in *Proceedings of the 43rd International ACM SIGIR Conference on Research and Development in Information Retrieval*, (New York, NY: United States Association for Computing), 1697–1700. 10.1145/3397271.3401214

[B10] Ge S.-G. Xia J. Sha W. Zheng C.-H. (2016). Cancer subtype discovery based on integrative model of multigenomic data. *IEEE ACM Trans. Comput. Biol. Bioinform.* 14 1115–1121. 10.1109/TCBB.2016.2621769 28113782

[B11] Holter N. S. Mitra M. Maritan A. Cieplak M. Banavar J. R. Fedoroff N. V. (2000). Fundamental patterns underlying gene expression profiles: simplicity from complexity. *Proc. Natl. Acad. Sci.* 97 8409–8414. 10.1073/pnas.150242097 10890920 PMC26961

[B12] Huang Y. Yuan K. Tang M. Yue J. Bao L. Wu S. (2020). Melatonin inhibiting the survival of human gastric cancer cells under ER stress involving autophagy and Ras-Raf-MAPK signalling. *J. Cell. Mol. Med.* 25 1480–1492. 10.1111/jcmm.16237 33369155 PMC7875909

[B13] Jia H. Ding S. Xu X. Nie R. (2014). The latest research progress on spectral clustering. *Neural Comput. Appl.* 24 1477–1486. 10.1007/s00521-013-1439-2

[B14] Jiang L. Wang C. Tang J. Guo F. (2019a). LightCpG: a multi-view CpG sites detection on single-cell whole genome sequence data. *BMC Genom.* 20:306. 10.1186/s12864-019-5654-9 31014252 PMC6480911

[B15] Jiang L. Xiao Y. Ding Y. Tang J. Guo F. (2019b). Discovering cancer subtypes via an accurate fusion strategy on multiple profile data. *Front. Genet.* 10:20. 10.3389/fgene.2019.00020 30804977 PMC6370730

[B16] Kong Y. Yu T. (2018). A graph-embedded deep feedforward network for disease outcome classification and feature selection using gene expression data. *Bioinformatics* 34 3727–3737. 10.1093/bioinformatics/bty429 29850911 PMC6198851

[B17] Lapointe J. Li C. Higgins J. P. Van De Rijn M. Bair E. Montgomery K. (2004). Gene expression profiling identifies clinically relevant subtypes of prostate cancer. *Proc. Natl. Acad. Sci.U.S.A.* 101 811–816. 10.1073/pnas.0304146101 14711987 PMC321763

[B18] Lee S. Lim S. Lee T. Sung I. Kim S. (2020). Cancer subtype classification and modeling by pathway attention and propagation. *Bioinformatics* 36 3818–3824. 10.1093/bioinformatics/btaa203 32207514

[B19] Li S. Jiang L. Tang J. Gao N. Guo F. (2020). Kernel fusion method for detecting cancer subtypes via selecting relevant expression data. *Front. Genet.* 11:979. 10.3389/fgene.2020.00979 33133130 PMC7511763

[B20] Liu K. Yang Y. (2018). Incorporating link information in feature selection for identifying tumor biomarkers by using miRNA-mRNA paired expression data. *Curr. Proteom.* 15 165–171. 10.2174/1570164614666171031160232

[B21] Mei S. Fei W. (2010). Amino acid classification based spectrum kernel fusion for protein subnuclear localization. *BMC Bioinform.* 11:S17. 10.1186/1471-2105-11-s1-s17 20122188 PMC3009488

[B22] Mootha V. K. Lindgren C. M. Eriksson K.-F. Subramanian A. Sihag S. Lehar J. (2003). PGC-1α-responsive genes involved in oxidative phosphorylation are coordinately downregulated in human diabetes. *Nat. Genet.* 34 267–273. 10.1038/ng1180 12808457

[B23] Pan X. Hu X. Zhang Y.-H. Chen L. Zhu L. Wan S. (2019). Identification of the copy number variant biomarkers for breast cancer subtypes. *Mol. Genet. Genom.* 294 95–110. 10.1007/s00438-018-1488-4 30203254

[B24] Pölsterl S. Gupta P. Wang L. Conjeti S. Katouzian A. Navab N. (2016a). Heterogeneous ensembles for predicting survival of metastatic, castrate-resistant prostate cancer patients. *F1000Research* 5:2676. 10.12688/f1000research.8231.3 28713544 PMC5500862

[B25] Pölsterl S. Navab N. Katouzian A. (2015). “Fast training of support vector machines for survival analysis,” in *Paper Presented at the Joint European Conference on Machine Learning and Knowledge Discovery in Databases*, eds Appice A. Rodrigues P. Santos Costa V. Gama J. Jorge A. Soares C. (Cham: Springer), 243–259. 10.1007/978-3-319-23525-7_15

[B26] Pölsterl S. Navab N. Katouzian A. (2016b). An efficient training algorithm for kernel survival support vector machines. *arXiv* [Preprint] arXiv:1611.07054

[B27] Rand W. M. (1971). Objective criteria for the evaluation of clustering methods. *J. Am. Statist. Assoc.* 66 846–850. 10.1080/01621459.1971.10482356

[B28] Schölkopf B. Smola A. Müller K.-R. (1998). Nonlinear component analysis as a kernel eigenvalue problem. *Neural comput.* 10 1299–1319. 10.1162/089976698300017467

[B29] Shen R. Olshen A. B. Ladanyi M. (2009). Integrative clustering of multiple genomic data types using a joint latent variable model with application to breast and lung cancer subtype analysis. *Bioinformatics* 25 2906–2912. 10.1093/bioinformatics/btp543 19759197 PMC2800366

[B30] Shen Y. Tang J. Guo F. (2019). Identification of protein subcellular localization via integrating evolutionary and physicochemical information into Chou’s general PseAAC. *J. Theor. Biol.* 462 230–239. 10.1016/j.jtbi.2018.11.012 30452958

[B31] Steinley D. (2004). Properties of the hubert-arable adjusted rand index. *Psychol. Methods* 9:386. 10.1037/1082-989X.9.3.386 15355155

[B32] Subramanian A. Tamayo P. Mootha V. K. Mukherjee S. Ebert B. L. Gillette M. A. (2005). Gene set enrichment analysis: a knowledge-based approach for interpreting genome-wide expression profiles. *Proc. Natl. Acad. Sci.U.S.A.* 102 15545–15550. 10.1073/pnas.0506580102 16199517 PMC1239896

[B33] Tomczak K. Czerwiñska P. Wiznerowicz M. (2015). The cancer genome atlas (TCGA): an immeasurable source of knowledge. *Contemp. Oncol.* 19:A68. 10.5114/wo.2014.47136 25691825 PMC4322527

[B34] Vert J.-P. Tsuda K. Schölkopf B. (2004). A primer on kernel methods. *Kernel Methods Comput. Biol.* 47 35–70. 10.7551/mitpress/4057.003.0004

[B35] Von Luxburg U. (2007). A tutorial on spectral clustering. *Statist. Comput.* 17 395–416. 10.1007/s11222-007-9033-z

[B36] Wang B. Mezlini A. M. Demir F. Fiume M. Tu Z. Brudno M. (2014). Similarity network fusion for aggregating data types on a genomic scale. *Nat. Methods* 11:333. 10.1038/nmeth.2810 24464287

[B37] Wang H. Ding Y. Tang J. Guo F. (2020). Identification of membrane protein types via multivariate information fusion with Hilbert-Schmidt independence criterion. *Neurocomputing* 383 257–269. 10.1016/j.neucom.2019.11.103

[B38] Yang Y. Huang N. Hao L. Kong W. (2017a). A clustering-based approach for efficient identification of microRNA combinatorial biomarkers. *BMC Genom.* 18:210. 10.1186/s12864-017-3498-8 28361698 PMC5374636

[B39] Yang Y. Xiao Y. Cao T. Kong W. (2017b). MiRFFS: a functional group-based feature selection method for the identification of microRNA biomarkers. *Int. J. Data Mining Bioinform.* 18 40–55. 10.1504/IJDMB.2017.10007184

[B40] Yeoh E.-J. Ross M. E. Shurtleff S. A. Williams W. K. Patel D. Mahfouz R. (2002). Classification, subtype discovery, and prediction of outcome in pediatric acute lymphoblastic leukemia by gene expression profiling. *Cancer Cell* 1 133–143. 10.1016/S1535-6108(02)00032-6 12086872

[B41] Yeung K. Y. Ruzzo W. L. (2001). Details of the adjusted rand index and clustering algorithms, supplement to the paper an empirical study on principal component analysis for clustering gene expression data. *Bioinformatics* 17 763–774. 10.1093/bioinformatics/17.9.763 11590094

