# Supplementary material for: Multi-Omics Data Fusion via a Joint Kernel Learning Model for Cancer Subtype Discovery and Essential Gene Identification
Source: Front Genet. 2021 Mar 4;12:647141. doi: 10.3389/fgene.2021.647141 (PMC7969795; doi:10.3389/fgene.2021.647141)
Supplement: Supplementary file 1 [file Data_Sheet_1.PDF]

Supplementary Material

1 Supplementary Tables

Supplementary Table1. Parameter γ on each data set.

**Datasets**	γ
**BRCA(4)**	1.40
**COAD(10)**	0.09
**KIDNEY(9)**	0.51
**LUNG(4)**	0.64
**STAD(6)**	0.09
**BLCA(9)**	0.12
**LIHC(7)**	1.57

2 Supplementary Figures

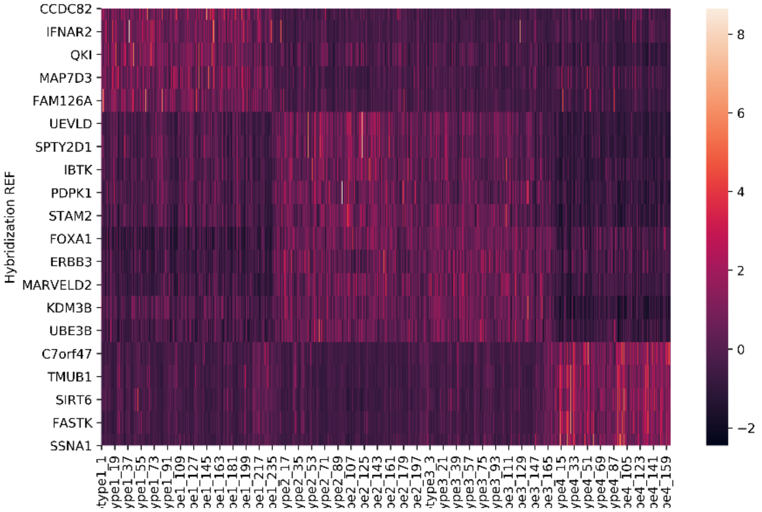

Supplementary Figure 1. Differential expression of some essential genes in different subtypes on BRCA.

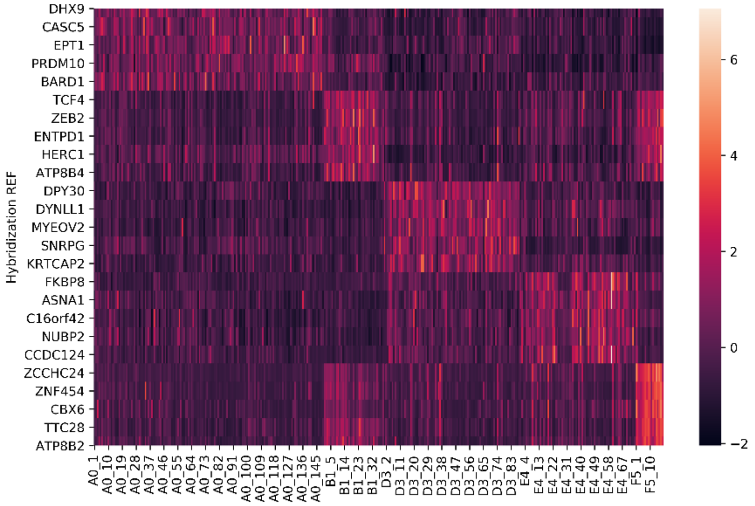

Supplementary Figure 2. Differential expression of some essential genes in different subtypes on STAD.

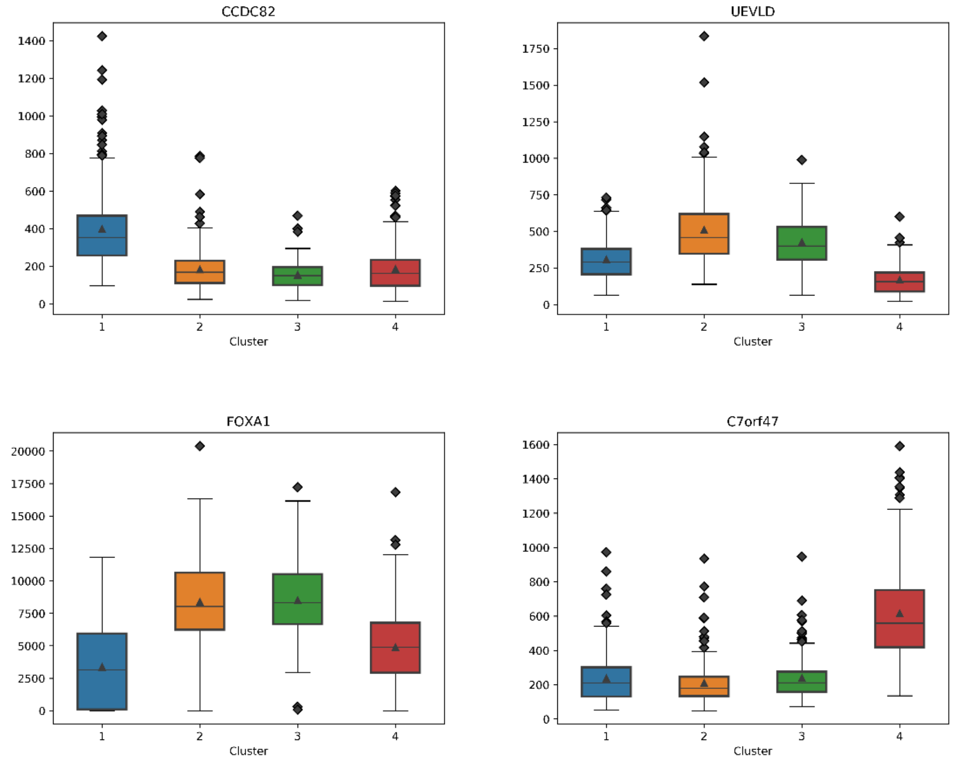

Supplementary Figure 3. The boxplots for essential genes supporting each subtype on BRCA.

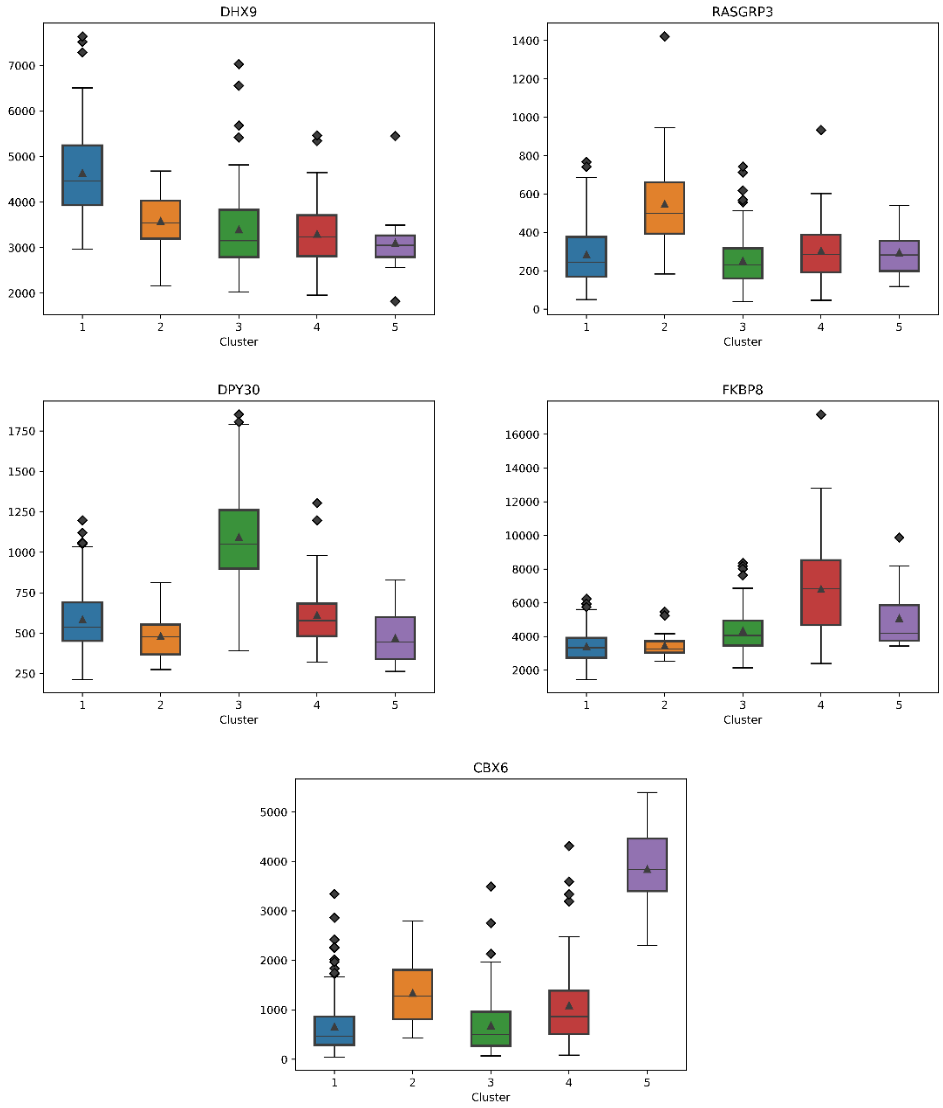

Supplementary Figure 4. The boxplots for essential genes supporting each subtype on STAD.
